# Microbe Profile: Planctopirus limnophila: the platypus of microbiology

**DOI:** 10.1099/mic.0.001731

**Published:** 2026-08-07

**Authors:** Elena Rivas-Marin, Damien P. Devos

**Affiliations:** 1 Departamento de Genética, Facultad de Biología, Universidad de Sevilla, Seville, Spain; 2 Institut Pasteur de Lille, Univ. Lille, CNRS, Inserm, CHU Lille, U1019-UMR 9017-Center for Infection and Immunity of Lille, F-59000 Lille, France

**Keywords:** bacterial endomembranes, budding, carotenoids, endocytosis, FtsZ-less, *Planctomycetota*

## GA legend

The pinkish bacterium *Planctopirus limnophila* displays unique features. Cells divide asymmetrically by budding, and the sacculi composition remains uncertain. The nucleoid is highly condensed, as evidenced by DAPI staining. Furthermore, these cells are capable of uptaking entire molecules, like GFP, into the periplasm for subsequent internal degradation. Many peculiarities of this organism are yet to be discovered or characterized.

## Taxonomy

Domain: *Bacteria*; superphylum: *Planctomycetota-Verrucomicrobiota-Chlamydiota* (PVC); phylum: *Planctomycetota*; class: *Planctomycetia*; order: *Planctomycetales*; family: *Planctomycetaceae*; genus: *Planctopirus*; species: *P. limnophila*. The genus includes three species (December 2025), and *P. limnophila* strain Mü 290T (DSM 3776=ATCC 43296) is the type strain of the genus.

## Properties

*P. limnophila* is a diderm, motile bacterium present in limnic environments. It was initially named *Planctomyces limnophilus* [1], where the species epithet indicates ‘lake-loving’ from the ‘Greek limnos’ (lake) and ‘philos’ (friend/loving). The genus name, however, indicates the initial confusion that surrounded the phylum since early on, from ‘planktos’ (wandering/floating) and ‘mukês’ (fungus), meaning floating fungi. The species is characterized by pink-to-red pigmentation, slow growth, low salt tolerance and an obligate aerobic metabolism. Its pear-shaped cells, with diameters ranging from 1.1 to 1.5 µm, possess a stalk composed of twisted fibrils. This stalk facilitates attachment to surfaces or other cells, frequently leading to rosette formation. It divides asymmetrically by budding, although other members of the phylum divide by binary fission or even alternate between both division modes. All planctomycetes lack the FtsZ protein.

## Genome

The genome consists of a single chromosome and one plasmid of 5,423,075 and 37,010 bp, respectively (GenBank Assembly Accession: GCA_000092105.1), with a G+C content of 53.5 mol% for the chromosome. Annotation identified a total of 4,131 genes, including 4,042 protein-coding sequences, along with 66 tRNA genes. Genomes available for *Planctomycetota*, including 172 from isolates and 2,138 metagenomes, present one of the highest proportions of genes with unknown functions among bacteria [2], underscoring the divergent, and mostly unknown, biology of the phylum. Specifically, in *P. limnophila*, 46.1% of its protein-coding genes lack annotation. Additionally, *P. limnophila* exhibits a high percentage of essential genes (18%), and 151 of them (out of 748, 20%) are of unknown function. Only a weak correlation is observed between gene conservation and essentiality [3]. Genetic manipulation in the phylum *Planctomycetota* is challenging, as they are slow-growing bacteria, and naturally resistant to most peptidoglycan (PG)-targeting antibiotics, due to the peculiar nature of their cell wall*.* However, *P. limnophila* is the strain most easily amenable to genetic manipulation and hence is the workhorse of this enigmatic phylum [3].

## Phylogeny and ecology

*P. limnophila* belongs to the *Planctomycetota* phylum, itself part of the PVC superphylum, which also encompasses *Verrucomicrobiota*, *Chlamydiota* and other less characterized phyla, such as *Lentisphaerota*, *Kiritimatiellota* and C*andidatus* Omnitrophota. This clade comprises phyla with diverse lifestyles, metabolisms and features, covering the full spectrum of symbiotic relationships, from mutualism to parasitism. Given this diversity, this grouping is unexpected, but supported by genomic analyses of conserved proteins, including housekeeping and ribosomal proteins. The current consensus firmly establishes the phylum *Planctomycetota* as a divergent lineage within the bacterial domain.

## Key features and discoveries

*Planctomycetota* is a peculiar phylum within the domain Bacteria, possessing characteristics that are rare in bacteria and sometimes more commonly associated with archaea or eukaryotes. Because of this, these bacteria have been nicknamed ‘the platypus of Microbiology’.

*P. limnophila* can metabolize a variety of complex carbon sources; however, very little is known about its functional metabolism.

Cell division is one of the most remarkable peculiarities of the phylum. The highly conserved FtsZ protein, the central bacterial division protein, is absent from all members of the *Planctomycetota* and *Chlamydiota* phyla. Within the phylum, species exhibit a variety of division modes, including binary fission, budding or even alternating between these modes; none of the underlying mechanisms have been characterized. In particular, *P. limnophila* divides by polar budding. Genomic analyses have revealed that most genes from the division and cell wall (*dcw*) cluster are non-essential, except for the one coding for the DNA pump FtsK. The non-essentiality of these genes, otherwise considered essential in most bacteria, suggests that this organism and other members of the phylum use novel division mechanisms based on non-canonical proteins [3].

Related to division, the presence and composition of PG within the *Planctomycetota* have been controversial since the initial isolations, with most strains exhibiting high resistance to all tested antibiotics targeting PG biosynthesis. They were initially described as lacking this universal bacterial structure, and their sacculi were subsequently thought to be predominantly proteinaceous. However, canonical PG components have since been detected in *P. limnophila* and others [4]. Despite these findings, the genes typically required for PG synthesis are not essential in this organism [3], and the precise composition of the sacculus remains unclear.

Another of the most distinctive features within the phylum, including *P. limnophila*, is the presence of a developed endomembrane system [5]. Strikingly, the nucleoid is abnormally condensed and occupies only a small portion of the cytoplasm within the internal membranes. Most planctomycetal genomes contain genes encoding proteins structurally related to eukaryotic membrane coat-like proteins, likely playing a role in the organization of the endomembrane system [6]. Linked to their endomembranes, endocytosis-like phenomena have been described [5, 7]. This phenomenon might be related to the phagocytosis-like process that internalizes whole cells detected in members of the class *Candidatus* Uabimicrobiia [8]. The molecular mechanisms behind these processes remain unknown.

Like most other Planctomycetes, *P. limnophila* synthesizes hopanoids as membrane regulators, but they are not essential [3]. This contrasts with the essentiality of a common hopanoid surrogate, sterol, in *Gemmata obscuriglobus*, another Planctomycete [9].

The characteristic pink-to-red pigmentation of the colonies is due to the production of C-30 carotenoids. These are produced by a novel and widespread pathway that uses squalene as a precursor [10], highlighting the novel biology found in this phylum.

These examples highlight some of the best-characterized cases, without overlooking the limited understanding and the many mysteries that remain hidden. More particularities are emerging as research on *P. limnophila*, in particular, and *Planctomycetota*, in general, progresses. This trend is further intensified by the ongoing isolation of novel species.

## Open questions

- As most genes from the *dcw* cluster are not conserved, and those that are are non-essential (with one exception), how does cell division occur without the universal FtsZ protein?
- As early studies claimed a proteinaceous content and most genes involved in PG synthesis are non-essential, what is the composition of the sacculi?
- Given that it represents one of the most developed prokaryotic endomembrane systems, what is the function of the planctomycetal endomembrane system?
- Since these are two unique processes in prokaryotes, what is the machinery responsible for endo- (and phago-) cytosis?
- As such diverse species, metabolism and features share a common origin, what are the evolutionary events that drove the diversity of the PVC superphylum?

## Abbreviations

*dcw*: division and cell wall
PG: peptidoglycan
PVC: *Planctomycetota-Verrucomicrobiota-Chlamydiota*
